# Dalbavancin in clinical practice in Spain: a 2 year retrospective study

**DOI:** 10.1093/jacamr/dlac120

**Published:** 2022-12-22

**Authors:** Laura Morata, José María Aguado, Miguel Salavert, Juan Pasquau, Enrique Míguez, Patricia Muñoz, Irantzu Rosselló, Benito Almirante

**Affiliations:** Service of Infectious Diseases, Hospital Clínic Barcelona, Calle de Villarroel, 170, Spain; Infectious Diseases Unit, Hospital Universitario 12 de Octubre, Avenida de Córdoba, s/n, Spain; Infectious Diseases Unit, Hospital Universitari i Politècnic La Fe, Avinguda de Fernando Abril Martorell, 106, Spain; Department of Infectious Diseases, Hospital Universitario Virgen de las Nieves, Avenida de las Fuerzas Armadas, 2, Spain; Division of Infectious Diseases, Hospital Universitario A Coruña, As Xubias 84, A Coruña 15006, Spain; Department of Medical Microbiology and Infectious Diseases, Hospital General Universitario Gregorio Marañón, Calle del Dr. Esquerdo, 46, Spain; Medical Department, Angelini Pharma España, Calle Antonio Machado, 78-80, Edificio Australia—3a planta, Viladecans 08840, Spain; Hospital Universitario Vall d’Hebrón, Passeig de la Vall d’Hebron, 119, Barcelona 08035, Spain; CIBERINFEC, ISCIII-CIBER de Enfermedades Infecciosas, Instituto de Salud Carlos III, Av. de Monforte de Lemos, 5, Madrid 28029, Spain

## Abstract

**Objectives:**

Dalbavancin is approved for the treatment of acute bacterial skin and skin-structure infections (ABSSSIs) in adults. Its unique pharmacokinetic properties allow daily dosing to be avoided. The objective was to describe the sociodemographic and clinical characteristics of patients treated with dalbavancin in Spain, and to evaluate its effectiveness and safety in real-world settings.

**Patients and methods:**

This non-interventional, retrospective, observational and multicentre study included patients who received at least one dose between 2018 and 2019 in seven Spanish hospitals.

**Results:**

In total, 187 patients were included. The most common comorbidities were cardiovascular disease (27.4%) and diabetes mellitus (23.5%). Dalbavancin was used to treat osteoarticular infections (28.3%), ABSSSIs (22.5%), cardiovascular infections (20.9%) and catheter-related infections (18.2%). The most prevalent pathogens were *Staphylococcus aureus* (34.2%), CoNS (32.6%), and enterococci (12.8%). The main reason for use was early hospital discharge (65.8%). Most patients were treated with 1500 mg in a single dose (35.3%) and the median duration of treatment was 2 weeks. The treatment was clinically successful in 91.4% of cases. Six patients (3.2%) reported adverse events. Physicians agreed on the potential reduction of hospitalization days (85.3%). A subanalysis of patient characteristics and type of pathogen showed similar results in terms of efficacy and safety.

**Conclusions:**

Dalbavancin seems to be effective and safe as second-line treatment in severe Gram-positive infections. It improves treatment adherence and allows outpatient management. Furthermore, the effectiveness and safety profile are maintained against diverse microorganisms in Gram-positive infections and regardless of the patients’ comorbidities at baseline, or age.

## Introduction

Over the past few years, a significant increase in the development of skin and soft tissue infections in healthcare settings has been reported.^1^ Resistant Gram-positive pathogens such as MRSA are associated with increased morbidity, mortality and healthcare expenditure in hospitalized patients. The incidence of MRSA is very relevant in many European countries, such as Romania (43.0%), Portugal (38.1%) and Greece (36.4%), and is 24.2% in Spain.^2^

In this context, vancomycin, daptomycin and linezolid are known to be active agents for the treatment of Gram-positive infections,^3–5^ but there are concerns regarding their toxicity, tolerance, drug resistance and patient adherence.^6–10^

Dalbavancin is a new lipoglycopeptide approved by EMA since 2015 for the treatment of acute bacterial skin and skin-structure infections (ABSSSIs) and is active against Gram-positive pathogens.^11,12^ Dalbavancin interferes in the formation of the bacterial cell wall by binding to the D-alanine-D-alanine terminal end of the peptidoglycan, halting its chain growth and ultimately resulting in bacterial cell death.^13^ Dalbavancin has unique pharmacokinetics properties, with a half-life of 14.4 days, permitting IV treatment of serious infections with one 1500 mg dose (infusion over 30 min), or 1000 mg followed 1 week later by 500 mg, without the need for daily dosing.^14,15^ Therefore, there is a decrease in both the need to maintain IV lines and the length of hospital stay, allowing early patient discharge and a reduction in high-cost hospital stays.^16–18^

An ABSSSI is defined as a bacterial infection of the skin such as cellulitis/erysipelas, major cutaneous abscess or wound infection, with a lesion size area of at least 75 cm^2^ (lesion size measured by the area of redness, edema or induration). The most common pathogens associated with ABSSSIs are Gram-positive bacteria such as *Staphylococcus aureus* and *Streptococcus pyogenes*. MRSA has become a leading cause of ABSSSIs, presenting a challenge for treatment.^19^

Randomized controlled trials (DISCOVER 1 and DISCOVER 2) have shown a favourable efficacy and safety profile of dalbavancin in patients with ABSSSIs.^20^ Dalbavancin has proven to be more active against MSSA, MRSA and glycopeptide-intermediate *S. aureus* when compared with other antibiotics.^21,22^ Dalbavancin was at least 16 times more potent than its comparators against all *S*. *aureus.* Furthermore, it had the lowest MIC for CoNS, followed by daptomycin, linezolid and vancomycin.

Although controlled clinical trials provide high-quality data of great validity, there is a need to obtain additional evidence from observational studies to confirm and define the characteristics of the drugs in real-world settings. In this regard, the clinical use of dalbavancin has extended to other Gram-positive infections [such as osteoarticular infections, infective endocarditis and catheter-related bloodstream infections (BSIs)], showing its efficacy and well-tolerated safety profile.^23–30^ Nevertheless, the available data are still scarce. Therefore, the objective of the present study was to describe the sociodemographic and clinical characteristics of the patients treated with dalbavancin in Spain, as well as to evaluate its effectiveness and safety in real-world settings.

## Materials and methods

### Ethics

The investigators and their collaborators committed to conduct the study in accordance with the International Council for Harmonisation (ICH) guidelines and guidelines for Good Clinical Practice (GCP), with the Declaration of Helsinki (revised version, Fortaleza, October 2013) and the local laws and guidelines of the countries in which the study was being conducted. The study was presented to the Ethics Committee for Investigation with medicinal products of the Vall d’Hebron University Hospital (Barcelona, Spain) and the approval was received on 12 March 2020. The initial approval obtained was then sent to the other Ethics Committees involved. The Spanish Agency for Medicines and Medical Devices (AEMPS) classified the study as an EPA-OD on 23 May 2019.

### Study design

This non-interventional, retrospective, observational and multicentre study included patients treated with dalbavancin in Spain between 2018 and 2019 (NCT04485676). Seven Spanish hospitals participated in the study: Hospital Vall d’Hebron (Barcelona), Hospital Clínic (Barcelona), Hospital 12 de Octubre (Madrid), Hospital La Fe (Valencia), Hospital Virgen de las Nieves (Granada), Hospital A Coruña (A Coruña) and Hospital Gregorio Marañón (Madrid). Inclusion criteria for patients were: male and female; adults (≥18 years old), patients who had received at least one dose of dalbavancin between 1 January 2018 and 31 December 2019; with available follow-up data for about 90 days after completing the treatment; and who had signed the written informed consent. The only exclusion criterion was participation in a clinical trial in which treatment with dalbavancin was managed through a protocol. The clinical management of patients and the treatment with dalbavancin was according to routine clinical practice.

### Endpoints and variables

The primary objective was the description of patient demographics and clinical characteristics. Secondary objectives included the effectiveness of dalbavancin, adverse events (AEs), treatment compliance assessment, the physician’s degree of satisfaction regarding the management of the infection with dalbavancin and the physician’s assessment of potential reduction in days of admission.

Effectiveness was evaluated regarding the time in days from administration to clinical response, which was evaluated at the end of treatment as per each investigator’s clinical judgement (success or failure), relapses and months of follow-up after last dose with dalbavancin with no relapses. Success of the treatment was defined as partial or complete resolution of leucocytosis, temperature and clinical signs or symptoms of infection. Failure of the treatment was defined as the persistence or progression of infection signs/symptoms, persistence of pathogens or growth of new microorganisms.

Safety was evaluated according to a description of single AE/adverse drug reaction (AE/ADR), severity, consideration of whether the AE was study drug-related or not (per assessment of investigator), start/stop date/time of any AE/ADR and any concomitant therapy to treat it (with start/stop date/time). Treatment discontinuation due to ADRs (number and reason) was also assessed.

Additionally, a subanalysis was performed considering patient characteristics (presence of diabetes mellitus, cardiovascular disease, aged over 60 years, and no presence of comorbidities at baseline) and the pathogen causing the infection. (*S. aureus* or enterococci). There was a subdivision regarding the type of infections. Cardiovascular infections were broken-down into right-sided endocarditis (pacemaker right-sided infective endocarditis and native valve right-sided infective endocarditis), left-sided endocarditis (prosthetic left-sided infective endocarditis and native left-sided infective endocarditis) and vascular prosthesis infection. Osteoarticular infection was divided into prosthetic and non-prosthetic infection (arthritis, spondylitis and osteomyelitis). Finally, catheter-associated infections were separated into complicated and uncomplicated bacteremia. Since publications on its use in non-approved indications are scarce, data relating to these subtypes of infections have been included in the Supplementary data, available at *JAC-AMR* Online).

### Determination of the sample size and data analysis

The sample size was calculated using variables (categorical or continuous) to which a cut-off point could be assigned or defined, allowing their dichotomous inclusion in the calculation. Conservatively, in accordance with the principle of maximum variance, a proportion of 50% was assumed in dichotomous variables. By considering a two-sided CI of 95% and assuming a sample proportion of 50%, a total sample size of 196 patients would provide a precision of ±7% to estimate the proportions for the primary objective. The calculations were made using the PASS software package, 2011 version. Categorical variables were described using absolute and relative frequencies, and continuous variables using the mean, median, SD and range (minimum–maximum values).

## Results

A total of 197 patients were initially included; however, 10 were excluded as they did not fulfil the study criteria. Therefore, 187 patients were finally analysed.

### Characteristics of patients

The following data are collected in Table 1. Patients were predominantly male (65.2%), with a mean age of 63.9 years. The most common comorbidities were cardiovascular disease (27.3%), diabetes mellitus (23.5%), solid tumour (15.0%) and chronic kidney disease (11.8%). The mean Charlson comorbidity index was 4.0 and the estimated 10 year survival was 46.4%.

**Table 1.** Sociodemographic and clinical characteristics of patients

	Patients (*N* = 187)
Age (years), mean (SD)	63.9 (18.6)
Gender, *n* (%)	
Male	122 (65.2)
Female	65 (34.8)
Comorbidities, *n* (%)	
None	27 (14.4)
Cardiovascular	51 (27.3)
Diabetes mellitus	44 (23.5)
Chronic kidney disease	22 (11.8)
Solid tumour	28 (15.0)
Respiratory disease	8 (4.3)
Leukaemia	7 (3.7)
Lymphoma	7 (3.7)
Metastatic solid tumour	4 (2.1)
Charlson comorbidity index, mean (SD)	4.0 (3.0)
Estimated 10 year survival, mean (SD)	46.4 (39.2)

### Type of infection and microorganisms

**Figure 1.**
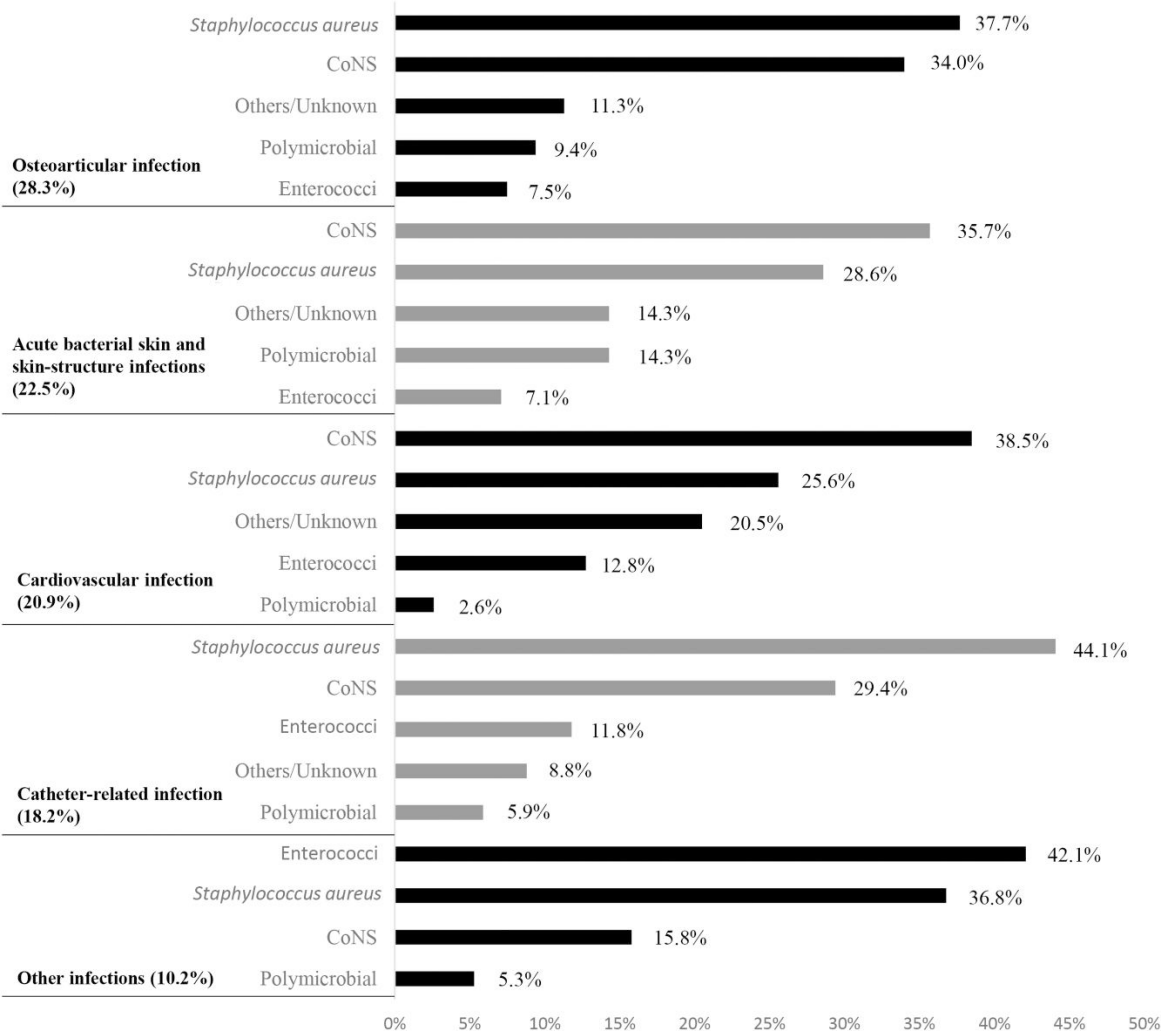
Type of infection and microorganisms.

The infections were related either to healthcare (62.0%) or were community acquired (patient had not recently been in a healthcare facility or been in contact with someone who had been recently in a healthcare facility, 34.8%). Dalbavancin was used to treat osteoarticular infections (28.3%), ABSSSIs (22.5%) and cardiovascular infections (20.9%), followed by catheter-related infections (18.2%) and other infections (10.2%, Figure 1, Table 2). The detailed data for cardiovascular, osteoarticular and catheter-related infections are shown in Table S1.

**Table 2.** Prior treatment and dalbavancin treatment characteristics (187 patients)

*N* (%)	Global population 187 (100.0)
Department that established treatment with dalbavancin, *n* (%)	
Infectious diseases	170 (90.9)
Internal medicine	10 (5.4)
Others	7 (3.7)
Prior antibiotic therapies	177 (94.7)
Number of therapies, *n* (%)	
1	41 (23.2)
2	58 (32.8)
3	35 (19.8)
≥4	43 (24.3)
Median number (range)	2.7 (1–10)
Antibiotic used, *n* (%)	
Daptomycin	103 (55.1)
Linezolid	37 (19.8)
Vancomycin	31 (16.6)
Time of prior antibiotic therapies, median days (range)	19 (1–360)
By type of infection, median (range)	
Osteoarticular infection	22 (3–300)
ABSSSI	18 (2–162)
Cardiovascular infections	28 (2–159)
Catheter-related bloodstream infection	14 (1–360)
Other infections	17.5 (1–56)
Control of the infection source, *n* (%)	113 (60.4)
Surgical drainage	47 (41.6)
Prosthesis removal	32 (28.3)
Catheter removal	27 (23.9)
Amputation/excision	6 (5.3)
Type of treatment, *n* (%)	
Targeted	176 (94.1)
Empirical	11 (5.9)
Reason for dalbavancin use, *n* (%)	
Early discharge	123 (65.8)
Previous treatment failure	21 (11.2)
Adverse event in previous treatment	9 (4.8)
Others/unknown	34 (18.2)
Duration of treatment (weeks), median (range)	2.0 (1–34)
Total number of doses, median (range)	1 (1–28)
Total dose administration (mg), median (range)	1500 (1000–27 000)
Compliance	181 (96.8)
Concomitant antimicrobial therapy, *n* (%)	61 (32.6)

The most common isolated pathogens were *S. aureus* (34.2% of patients, 59.43% being MSSA and 31.3% MRSA), CoNS (32.6%, mainly *Staphylococcus epidermidis*, in 65.6% of them) and *Enterococcus* spp. (12.8%). The microorganism responsible for each type of infection is shown in Figure 1.

There was adequate control on the focus of the infection in 60.4% patients, mainly by surgical drainage (41.6%), prosthesis removal (28.3%) or catheter removal (23.9%).

### Prior antibiotic therapy

The following data are collected in Table 2. Most patients had been previously treated with other antibiotics (94.7%) for a median duration of 19 days. The mean number of previous treatments was 2.7. The majority of patients had been treated with daptomycin (55.1%), linezolid (19.8%) and vancomycin (16.6%).

### Dalbavancin treatment

The data regarding the therapy are collected in Table 2, and Table S1 for cardiovascular, osteoarticular and catheter-related infections. The treatment with dalbavancin was mostly established by the infectious diseases department (90.9%). It was predominantly targeted (94.1%), with a median duration of 2.0 weeks (range: 1–34). The median number of doses was 1 (range: 1–28) with a total median administration of 1500 mg (range: 1000–27 000). The maximum dose was administered to only one patient on suppressive treatment, who was indicated for surgery that could not be performed due to the unacceptable risk to life. The case was a 53-year-old man with diabetes and cardiovascular disease with endocarditis in aortic mechanical prosthesis and Dacron aortic tube. The *Streptococcus gallolyticus* infection was first treated with linezolid, and then switched to dalbavancin to facilitate patient discharge. The patient started the treatment with dalbavancin in July 2018 with a posology of 1500 mg + 1500 mg/2 weeks (17 doses) with a duration of 34 weeks and a total dose of 27 000 mg. There was clinical response, without relapses. He had an AE of mild asthenia in August 2018 and without sequelae that ended in March 2019.

**Figure 2.**
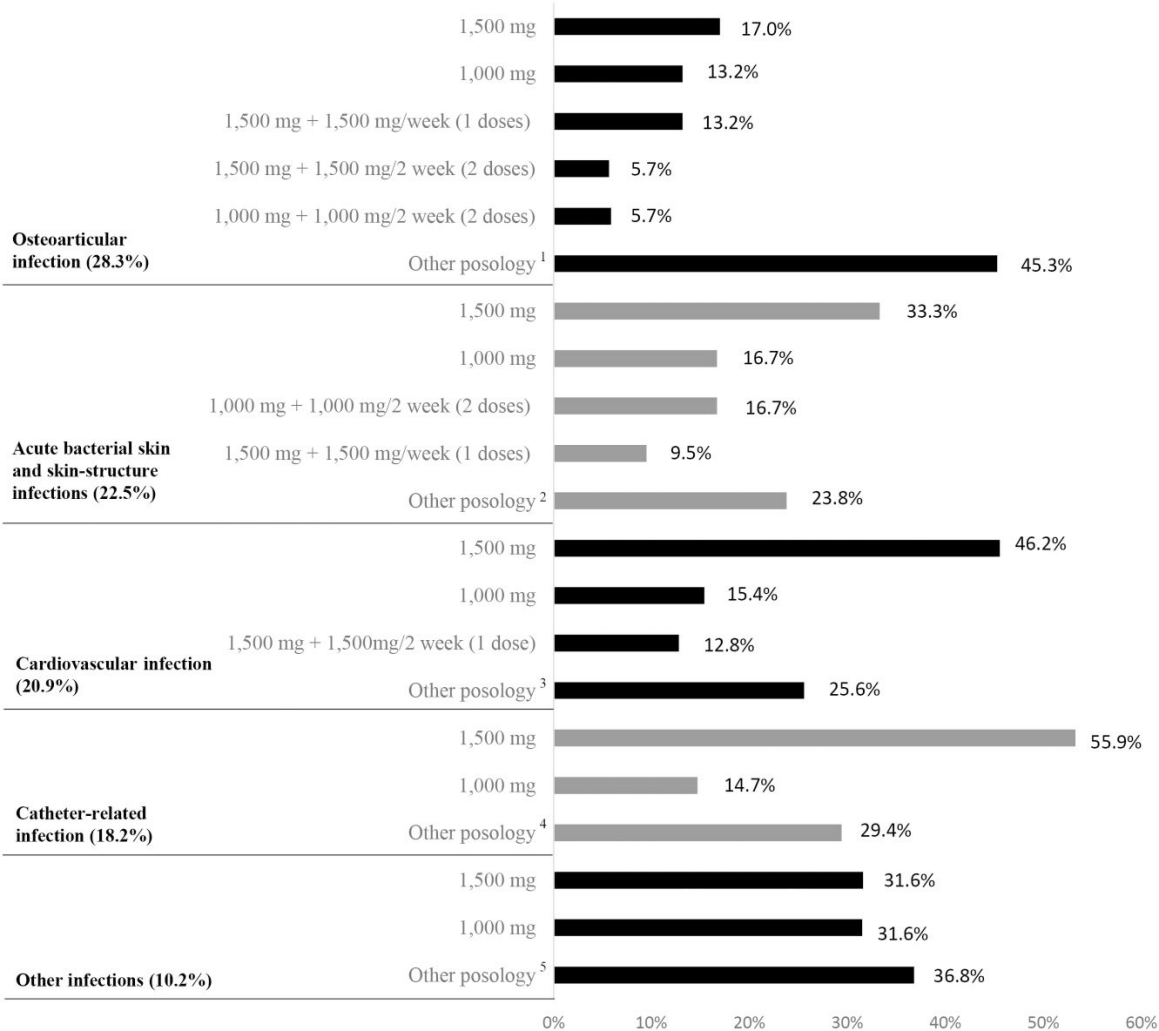
Detailed dosing per type of infection. Note 1: the longest posology was 1500 mg + 1500 mg/2 weeks (11 doses); note 2: the longest posology was 1000 mg + 500 mg/weeks (10 doses); note 3: the longest posology was 1500 mg + 1500 mg/2 weeks (17 doses); note 4: the longest posology was 1000 mg + 1500 mg/2 weeks (3 doses); note 5 the longest posology was 1000 mg + 1000 mg/2 weeks (5 doses).

The detailed dosing per type of infection is shown in Figure 2. The main reason for dalbavancin use was early hospital discharge (65.8%), followed by previous treatment failure (11.2%) and adverse reaction (4.8%). Concomitant antimicrobial therapy with the dalbavancin treatment was received by 32.6% of patients. The most commonly administered treatment was rifampicin (16.4%), followed in frequency by ciprofloxacin (13.1%). Up to 96.8% of patients complied with the treatment.

### Effectiveness of dalbavancin

**Figure 3.**
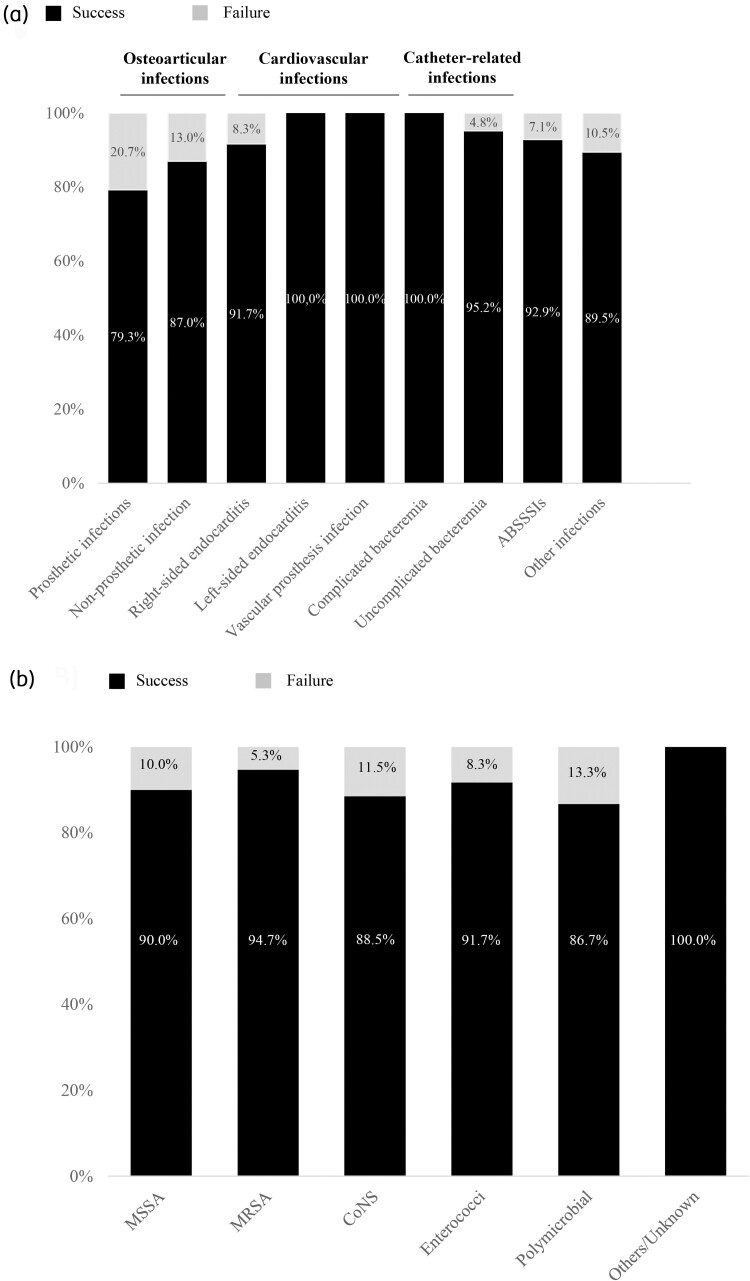
Treatment outcomes by infection type (a) or by aetiology (b).

The detail of success for each type of infection or microorganism is described in Figure 3. The treatment with dalbavancin was considered successful in 91.4% of the patients. The mean time from treatment onset to clinical response was 9.3 days.

In total, 8.6% patients had a failure due to relapse (6.5%) or other causes (2.1%). Detailed data are shown in Table S2. Mean time to relapse from the last dose of dalbavancin was 1.7 months; mean time of follow-up after the last dose of dalbavancin with no relapses was 7.0 months (range: 0–32). Additionally, 5.3% patients who did not receive prior antibiotic therapy had comparable efficacy and safety results (Table S3).

### Safety profile

The details regarding the tolerability and safety profile are described in Table 3. AEs related to dalbavancin were experienced by 3.2% of patients and only 1.1% discontinued the treatment due to AEs related to dalbavancin.

**Table 3.** Safety of dalbavancin treatment

	Patients (*N* = 187)
Adverse events^a^, *n* (%)	6 (3.2)
Intensity, *n* (%)	
Mild	4 (66.7)
Moderate	1 (16.6)
Severe	1 (16.6)
Related to dalbavancin, *n* (%)	
Possible	4 (66.7)
Probable	2 (33.3)
Treatment discontinuation due to adverse events^b^, *n* (%)	2 (1.1)

a Mild testicular oedema (*n* = 1), mild dizziness (*n* = 1), mild pruritus (*n* = 1), mild asthenia (*n* = 1), moderate balanitis (*n* = 1), severe thrombopenia (*n* = 1).

b Reasons: testicular oedema, severe thrombopenia.

### Physicians’ opinion on infection management with dalbavancin

**Figure 4.**
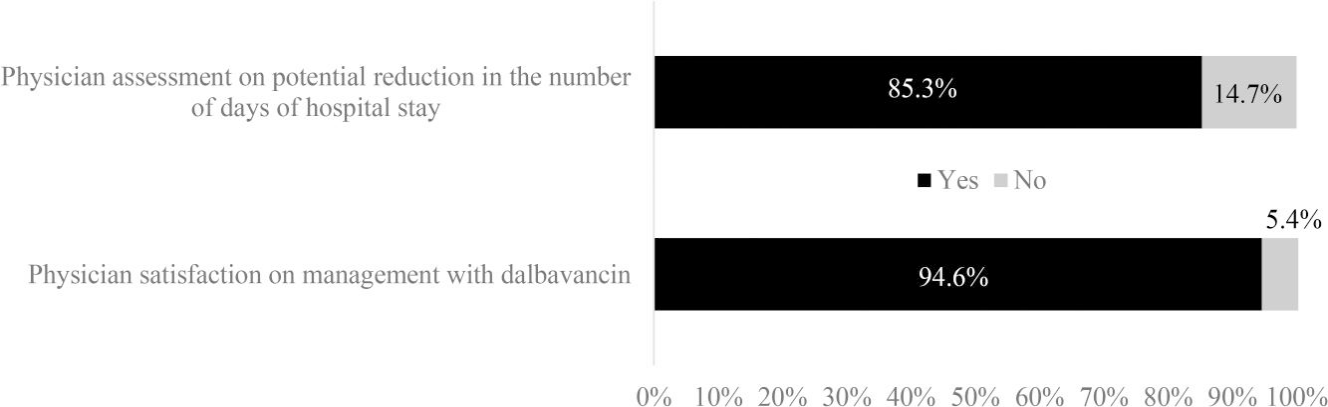
Physician’s opinion on infection management with dalbavancin.

Most physicians agreed on the potential reduction in the number of hospitalization days (85.3%, Figure 4). Also, the satisfaction with dalbavancin was high (94.6%).

### Subanalysis according to patient characteristics

In this subanalysis, the total population was divided into four subgroups: patients with diabetes mellitus; cardiovascular disease; over 60 years old; and with no comorbidities at baseline. The outcomes for these subgroups are described in Table 4 and detailed data are shown in Table S4. In general, the majority of patients were treated with dalbavancin due to early patient discharge. The most common types of infection were osteoarticular infections, ABSSSIs and cardiovascular infections. The microorganisms with higher prevalence were *S. aureus*, CoNS and enterococci. The clinical response was high and low relapse rates were observed.

**Table 4.** Clinical success and safety by patient subgroups

	Number of patients (*N*)	Clinical success (%)	Relapses (%)	AE (%)
Overall	187	91.4	6.5	3.2
Diabetes	44	88.6	9.1	2.3
Cardiovascular disease	51	94.0	3.9	3.9
Over 60 years	113	91.2	6.2	0.9
No comorbidities at baseline	27	96.3	3.7	7.4
*S. aureus* infection	64	92.2	6.3	3.1
*Enterococcus* infection	24	91.7	8.3	0.0

### Subanalysis by type of microorganism pathogen

Two subgroups were analysed, namely *S. aureus* and enterococci infections. The main outcomes for each are described in Table 4, and the detailed data are shown in Table S5. The main reason for use was early patient discharge. Regarding the type of infections, the most common ones were osteoarticular, ABSSSIs, cardiovascular and catheter-related BSIs. In the *S. aureus* subgroup, MSSA was the most prevalent. Among the enterococci subgroup, the percentages of *Enterococcus faecalis* and *Enterococcus faecium* were similar. The clinical response was higher in the *S. aureus* subgroup than the enterococci subgroup, with similar relapse rate.

## Discussion

Dalbavancin was approved for the treatment of ABSSSIs caused by Gram-positive bacteria.^11,31^ However, literature showed it being administered beyond the label indication in Spain and in other countries.^23–30^ The first published results of dalbavancin in a real-world setting in Spain showed its use during 2016 and 2017. This retrospective study included 69 patients from 29 hospitals.^23^ Subsequently, the effectiveness of dalbavancin was evaluated in 83 patients diagnosed with BSI and/or endocarditis in Spanish hospitals.^25^ Of these patients, 49% had *S. aureus* in BSI, and 44.1% presented with CoNS in endocarditis. All the patients achieved clinical cure in the hospital, with no relapses 3 months later in the case of BSI. A Spanish multicentre study evaluated the response in 64 patients diagnosed with osteoarticular infection.^28^ The most prevalent microorganisms were *S. epidermis* (46.9%) and *S. aureus* (21.9%). Patients received dalbavancin due to simplification of the regimen or AEs. In 23 out of 45 patients with infection associated with the orthopaedic implant, it was preserved. In this subgroup, 65.2% were cured and 34.8% showed improvement. Another multicentre retrospective study assessed the effectiveness and safety profile of dalbavancin in 36 patients with osteomyelitis.^26^ Here, 90% achieved clinical success, without reported AEs.

Our study reflects the use of dalbavancin in recent years. It enables an analysis to be done of the evolution of its use in clinical practice since its marketing authorization in Spain. In our study, the clinical and demographic characteristics were similar to those of Bouza *et al*.^23^ A comparison of the comorbidity profile showed the median Charlson comorbidity index as 3 in the previous study while the mean value was 4 in our analysis. The most common underlying diseases in both studies were diabetes mellitus (33.3% versus 23.5%, respectively) and cardiovascular disease (31.9% versus 27.3%). However, in our study, chronic kidney disease (11.8%) and solid tumour (15.0%) were some of the most prevalent diseases but in the study by Bouza *et al.*, respiratory tract disease (21.7%) and neurological disorder (20.3%) were more abundant. In both studies, the vast majority of patients had received prior antibiotic therapy (97.1% versus 94.7%, respectively). Bouza *et al.* found the median number of antibiotics prior to dalbavancin to be 2, and the mean figure in our study was 2.7. The main reason for use was the same in both studies (easier antibiotic administration and early patient discharge, respectively). Nevertheless, there are differences in the use of dalbavancin. Regarding the type of infection, the main usage in the previous study and in ours was for osteoarticular infection (46.4% versus 28.3%, respectively), ABSSSIs (21.7% versus 22.5%), catheter-related infection (11.6% versus 18.2%) and endocarditis (10.1% versus 20.9%). Focusing on the type of microorganisms, the trends in Bouza *et al.*’s study and ours were as follows: MRSA (26.2% versus 20.3%, respectively), MSSA (18.0% versus 10.7%), CoNS (39.3% versus 32.6%) and enterococci (21.3% versus 12.8%). In our analysis, the median duration of treatment with dalbavancin was decreased by 1 week (2.0 weeks versus 21 days), while the mean concomitant antimicrobial therapy was increased (39.4 versus 25 days). Similar to previous findings, dalbavancin was successful not only for the approved indication in ABSSSIs but also for other infections, at even higher rates, with a global efficacy of 91.4% versus 84.1% in Bouza *et al.*’s study. According to infection type, dalbavancin showed its effectiveness in ABSSSIs (80% versus 92.9%, respectively), endocarditis (85.7% versus 97.4%), catheter-related infection (75% versus 97.1%), osteoarticular infection (80% prosthetic joint infection and 91.7% osteomyelitis versus 82.7%). Finally, in Bouza *et al.*’s study, AEs were either mild (10.1%) or severe (2.9%), while in ours only 3.2% of patients reported AEs and the majority (66.7%) were mild. The safety profile seemed more favourable in our study, with low rates of discontinuation, which could be related to a broader knowledge and management of the drug over the years.

Another relevant issue is the potential reduction in length of stay and potential cost saving related to dalbavancin use. This was retrospectively assessed in 50 patients with Gram-positive infections (2017–19).^18^ After 14 days of a prior antimicrobial drug, the treatment was switched to dalbavancin. By switching medication, the saving amounted to €8259 and 14 days of hospitalization per patient. A similar study was conducted in Spain.^17^ The administration of dalbavancin provided €4550 savings per patient. In our study, an economic analysis was not performed. However, data regarding the physician’s opinion on the reduction of the hospitalization stay were collected. Up to 85.3% of the physicians agreed on the potential reduction in days of admission, with the additional benefit of decreasing the risk of nosocomial complications as well as the healthcare costs.

Regarding the safety profile, a narrative review found no significant differences between dalbavancin and comparators in terms of incidence of AEs.^32^ Moreover, a posterior systematic review and meta-analysis was performed regarding randomized clinical trials.^33^ In total, 30.6% of patients experienced AEs related to dalbavancin (4.80% were severe), and there was a lower mortality rate. There are papers addressing the safety profile in clinical practice. In a Spanish study, AEs occurred in 3.9% of the patients, with the most prevalent being skin rash, nausea and vomiting, infusion reaction and hypersensitivity.^17^ Out of 102 patients, only one discontinued due to AEs. In another study with 64 patients, there were seven AEs (gastrointestinal problems, phlebitis, asthenia, rash, increase of serum creatinine), but no patient discontinued.^28^ Other authors showed that only 4.8% experienced AEs and none caused discontinuation.^25^ In Austria, another study evaluated the efficacy and safety to treat endocarditis in 27 patients.^24^ Of these, two presented AEs (nausea/vomiting and increase in serum creatinine) and one discontinued the treatment due to failure. Our findings coincide with the general safety profile, characterized by a low incidence of AEs (3.2%), which are predominantly mild (66.7%) and that do not directly require discontinuation (1.1%). Finally, in our study, up to 32.6% of patients received concomitant antimicrobial therapy. *In vitro* receptor screening studies indicate that dalbavancin is unlikely to interact with other therapeutic targets or show potential for relevant pharmacodynamic interactions.^12^

Moreover, our subanalysis based on patient characteristics and infection type highlight the similar efficacy and safety regardless of the comorbidities at baseline and in the management of different pathologies. Furthermore, data from our study show that dalbavancin can be used in indications other than those approved for its clinical use, even in patients with significant comorbidities, with an excellent efficacy and safety profile.

Finally, our study is the first to evaluate treatment adherence (96.8%). Ensuring compliance is of utmost importance, as patient adherence to oral antibiotic therapy after hospital discharge is low and associated with a poor clinical outcome.^10^ Additionally, the study assessed the physician’s satisfaction with the infection management, and this was generally high (94.6%).

The main limitation of the study is its intrinsic retrospective nature and relatively small sample size. Nevertheless, much information was retrieved from the medical records of patients. Another limitation was the variability between data collectors across centres, which was addressed by data quality assurance, including on-site and remote monitoring, among other measures.

### Conclusions

Dalbavancin seems to be effective and safe as second-line treatment in severe infections caused by Gram-positive pathogens, above and beyond ABSSSIs (basically cardiovascular infections and osteoarticular infections). Dalbavancin improves adherence and allows outpatient management, reducing hospital stay and potential associated risks. Furthermore, administration with different dosage schedules offers an optimal safety profile maintaining the effectiveness and safety profile regardless of the patients’ comorbidities at baseline or age.

## Supplementary Material

dlac120_Supplementary_Data

## Transparency declarations

L. Morata has received honoraria for talks and lectures from Merck Sharp and Dohme (MSD) and Angelini Pharma. J. M. Aguado has received honoraria for consultancy and participated in speakers’ bureaus from Angelini Pharma, Astellas Pharma, Pfizer, Gilead, MSD, United Medical and Roche. M. Salavert has received honoraria for participating in scientific consultancies on antimicrobials and lecturing at meetings promoted by the following pharmaceutical companies in the last 4 years: Angelini Pharma, Gilead, GlaxoSmithKline, Janssen, Menarini, MSD, Shionogi and Pfizer. J. Pasquau has received honoraria for talks, consultancy, educational events or research support from Pfizer, MSD, Angelini Pharma, Menarini, Advanz Pharma and Astellas. E. Míguez has received honoraria for talks from Pfizer, MSD, Shionogi and Menarini. P. Muñoz has received honoraria for consultancy and lectures from Angelini Pharma, Gilead, MSD, Pfizer, Roche and Werfen, as well as from scientific societies and non-profit foundations, such as Fundación de Ciencias de la Salud, UIMP and Future Day Foundation. P. Muñoz has also received honoraria for research from FIS, CIBER Enfermedades Respiratorias, REIPI, Mutua Madrileña, European Community Funds and Fundación del Pino. I. Rosselló is employed by Angelini Pharma. B. Almirante has received honoraria for speaking at educational events from MSD, Pfizer Inc., Gilead Sciences and Angelini Pharma, and has received research funding from Gilead Sciences, MSD and Pfizer Inc.

## Supplementary data

Tables S1 to S5 are available as Supplementary data at *JAC-AMR* Online.
