# Supplementary material for: Dalbavancin in clinical practice in Spain: a 2 year retrospective study
Source: JAC Antimicrob Resist. 2022 Dec 22;4(6):dlac120. doi: 10.1093/jacamr/dlac120 (PMC9777743; doi:10.1093/jacamr/dlac120)
Supplement: dlac120_Supplementary_Data [file dlac120_supplementary_data.docx]

**Table S1**. Prior treatment and dalbavancin treatment characteristics for cardiovascular, osteoarticular and catheter-related infections

| N (%) | Cardiovascular infection  39 (20.9) | | | Osteoarticular infection  53 (28.3) | | Catheter-related infection  34 (18.2) | | |
| --- | --- | --- | --- | --- | --- | --- | --- | --- |
|  | Right-sided endocarditis  12 (30.8) | Left-sided endocarditis  26 (66.6) | Vascular prosthesis infection  1 (2.6) | Prosthetic infection  29 (54.7) | Non-prosthetic infection  24 (45.3) | Complicated bacteremia  13 (38.2) | Uncomplicated bacteremia  21 (61.8) | |
| Department that established treatment with dalbavancin, n (%) | | | | | | | | |
| Infectious diseases | 11 (91.7) | 25 (96.2) | 1 (100.0) | 22 (75.9) | 22 (91.6) | 12 (92.3) | 17 (81.0) | |
| Internal Medicine | 0 (0.0) | 1 (3.8) | 0 (0.0) | 2 (6.9) | 1 (4.2) | 0 (0.0) | 1 (4.8) | |
| Others | 1 (8.3) | 0 (0.0) | 0 (0.0) | 5 (17.2) | 1 (4.2) | 1 (7.7) | 3 (14.2) | |
| Prior antibiotic therapies | 11 (91.7) | 25 (96.2) | 1 (100.0) | 27 (93.1) | 23 (95.8) | 13 (100.0) | 20 (95.2) | |
| Median number (range) | 2 (1-4) | 3 (1-10) | 3 | 2.3 (1-7) | 2.7 (1-10) | 3 (1-6) | 2 (1-5) | |
| Antibiotic used, n (%) | | | | | | | | |
| Daptomycin | 7 (63.6) | 16 (64.0) | 1 (100.0) | 15 (55.6) | 14 (60.9) | 9 (69.2) | 15 (75.0) | |
| Linezolid | 0 (0.0) | 6 (24.0) | - | 6 (22.2) | 4 (17.4) | - | - | |
| Vancomycin | - | - | 1 (100.0) | - | - | 4 (30.8) | 4 (20.0) | |
| Control of the infection source, n (%) | 8 (66.7) | 6 (23.1) | 1 (100.0) | 23 (79.3) | 15 (62.5) | 7 (53.8) | 18 (85.7) | |
| Surgical drainage | 0 (0.0) | 1 (16.6) | 1 (100.0) | 11 (47.8) | 11 (73.3) | - | - | |
| Prosthesis removal | 7 (87.5) | 5 (83.4) | 0 (0.0) | 12 (52.2) | 2 (13.3) | - | - | |
| Catheter removal | 1 (12.5) | 0 (0.0) | 0 (0.0) | - | - | 7 (100.0) | 18 (100.0) | |
| Amputation/Excision | 0 (0.0) | 0 (0.0) | 0 (0.0) | 0 (0.0) | 2 (13.3) | - | - | |
| Type of treatment, n (%) | | | | | | | |  |
| Targeted | 10 (83.3) | 25 (96.2) | 1 (100.0) | 28 (96.6) | 22 (91.7) | 12 (92.3) | 21 (100.0) | |
| Empirical | 2 (16.7) | 1 (3.8) | 0 (0.0) | 1 (3.4) | 2 (8.3) | 1 (7.7) | 0 (0.0) | |
| Reason for dalbavancin use, n (%) | | | | | | | |  |
| Early discharge | 7 (58.3) | 23 (88.5) | 0 (0.0) | 16 (55.2) | 14 (58.3) | 9 (69.2) | 13 (61.9) | |
| Previous treatment failure | 1 (8.4) | 2 (7.7) | 1 (100.0) | 10 (34.5) | 7 (29.2) | 2 (15.4) | 1 (4.8) | |
| Adverse event in previous treatment | - | - | 0 (0.0) | - | - | - | - | |
| Others/Unknown | 4 (33.3) | 1 (3.8) | 0 (0.0) | 3 (10.3) | 3 (12.5) | 2 (15.4) | 7 (33.3) | |
| Duration of treatment, median weeks (range) | 2.5  (1.0-6.0) | 2.0  (1.0-34.0) | 4.0 | 4 (2-6) | 2 (1-5) | 1 (1-4) | 1 (1-11) | |
| Total number of doses, median (range) | 1 (1-3) | 1 (1-3) | 3 | 2 (2-4) | 2 (1-4) | 1 (1-3) | 1 (1-4) | |
| Total dose administration, median mg (range) | 1,500 (1,000-4,500) | 1,500 (1,000-27,000) | 3,500 | 3,000 (1,000-18,000) | 2,750 (1,000-13,500) | 1,500 (1,000-4,500) | 1,500 (1,000-5,500) | |
| Compliance | 12 (100.0) | 25 (96.2) | 1 (100.0) | 28 (96.6) | 24 (100.0) | 13 (100.0) | 21 (100.0) | |
| Concomitant antimicrobial therapy, n (%) | 3 (25.0) | 4 (15.4) | 0 (0.0) | 11 (37.9) | 12 (50.0) | 2 (15.4) | 5 (23.8) | |
| Micro-organisms | | | | | | | | |
| *Staphylococcus aureus* | 3 (25.0) | 7 (26.9) | 0 (0.0) | 11 (37.9) | 9 (37.5) | 5 (38.5) | 10 (47.6) | |
| MSSA | 2 (66.7) | 5 (71.4) | 0 (0.0) | 2 (18.2) | 7 (77.8) | 5 (100.0) | 9 (90.0) | |
| MRSA | 1 (33.3) | 2 (28.6) | 0 (0.0) | 7 (63.6) | 2 (22.2) | 0 (0.0) | 1 (10.0) | |
| Unknown | - | - | 0 (0.0) | 2 (18.2) | 0 (0.0) | - | - | |
| CoNs | 4 (33.3) | 10 (38.5) | 0 (0.0) | 9 (31.0) | 9 (37.5) | 3 (23.1) | 7 (33.3) | |
| *Staphylococcus epidermidis* | 2 (50.0) | 7 (70.0) | 1 (100.0) | 9 (100.0) | 4 (44.4) | 2 (66.7) | 2 (28.6) | |
| Others | 2 (50.0) | 3 (30.0) | 0 (0.0) | 0 (0.0) | 5 (55.6) | 1 (33.3) | 5 (71.4) | |
| Enterococci | 0 (0.0) | 5 (19.2) | 0 (0.0) | 3 (10.3) | 1 (4.2) | 2 (15.4) | 2 (9.5) | |
| *Enterococcus faecalis* | 0 (0.0) | 2 (40.0) | 0 (0.0) | 3 (100.0) | 1 (100.0) | 1 (50.0) | 1 (50.0) | |
| *Enterococcus faecium* | 0 (0.0) | 3 (60.0) | 0 (0.0) | - | - | 1 (50.0) | 1 (50.0) | |
| Vancomycin-susceptible | 0 (0.0) | 4 (80.0) | 0 (0.0) | 3 (100.0) | 1 (100.0) | 1 (50.0) | 2 (100.0) | |
| Polymicrobial | 1 (8.3) | 0 (0.0) | 0 (0.0) | 4 (13.8) | 1 (4.2) | 2 (15.4) | 0 (0.0) | |
| Others/Unknown | 4 (33.3) | 4 (15.4) | 0 (0.0) | 2 (6.9) | 4 (16.7) | 1 (7.7) | 2 (9.5) | |
| Posology | | | | | | | | |
| 1,500 mg | 5 (41.7) | 13 (50.0) | 0 (0.0) | 5 (17.2) | 4 (16.7) | 7 (53.8) | 12 (57.2) | |
| 1,000 mg | 3 (25.0) | 3 (11.5) | 0 (0.0) | 2 (10.3) | 5 (20.8) | 3 (23.1) | 2 (9.5) | |
| 1,500 mg + 1,500 mg/2 week (1 doses) | 2 (16.7) | 3 (11.5) | 0 (0.0) | 4 (13.8) | 3 (12.5) | - | - | |
| 1,500 mg + 1,500 mg/week (1 doses) | - | - | 0 (0.0) | - | - | - | - | |
| 1,500 mg + 1,000 mg/2 week (2 doses) | - | - | 1 (100.0) | - | - | - |  | |
| Other posology | 2 (16.7) | 7 (27.0) | 0 (0.0) | 18 (62.1) | 12 (50.0) | 3 (23.1) | 7 (33.3) | |

**Table S2**. Clinical characteristics of treatment failure

| **Age** | **Sex** | **Type of infection** | **Infection focus** | **Control source** | **Reason for dalbavancin use** | **Microorganism** | **Previous antimicrobial therapy (days)** | **Dalbavancin treatment** | **Cause of failure** |
| --- | --- | --- | --- | --- | --- | --- | --- | --- | --- |
| 29 | Female | Other infection  (Pancreatitis) | Health care | Surgical drainage | Early patient discharge | *Staphylococcus epidermidis* | Three treatments used for 19 days: cotrimoxazol (7), linezolid (4), ertapenem (8) | 1.000 mg as single dose  Concomitant antimicrobial therapy | Clinical and microbiological reasons |
| 56 | Female | Osteoarticular infection  (Non prosthetic infection: septic arthritis) | Health care | Surgical drainage | Adverse reaction of previous treatment | *Staphylococcus lugdunensis* | Seven treatments used for 35 days: piperacillin/tazobactam (2), levofloxacin (4), meropenem (6), levofloxacin (1), ceftriaxone (3), daptomycin (13), colistin (6) | 1,000 mg + 1,000 mg/2 weeks (3 doses)  Concomitant trimetroprim-sulfametoxazol | Relapse |
| 46 | Male | Osteoarticular infection  (Prosthetic infection) | Health care | Surgical drainage | Early patient discharge | MRSA | Four treatments used for 22 days: ceftazidime (7), ciprofloxacin (6), daptomycin (7), ciprofloxacin (2) | 1,000 mg + 500 mg/week (5 doses) + 1,000 mg/2 weeks (2 doses) | Relapse |
| 49 | Female | ABSSSI  (Wound infection) | Health care | Surgical drainage | Early patient discharge | *Staphylococcus epidermidis* | Two treatments used for 9 days: ceftazidima (4), vancomicina (5) | 1,000 mg + 1,000 mg/week (1 dose)  Concomitant antimicrobial therapy | Relapse |
| 58 | Male | Osteoarticular infection  (Non prosthetic infection: septic arthritis) | Community-acquired | Surgical drainage | Simplification of treatment | MSSA | Three treatment used for 62 days: cloxacillin (31), daptomycin (4), levofloxacin (27) | 1,000 mg as single dose  Concomitant antimicrobial therapy (levofloxacin) | Relapse |
| 74 | Male | Other infection  (Cholangitis) | Health care | No | Unknown reason | *Enterococcus faecium* | Five treatments used for 29 days: meropenem (3), teicoplanin (10), ciprofloxacin (12), teicoplanin (2), ciprofloxacin (2) | 1,000 mg + 1,000 mg/2 weeks (2 doses)  Concomitant antimicrobial therapy (ciprofloxacin) | Relapse |
| 89 | Male | Osteoarticular infection  (Prosthetic infections) | Health care | Surgical drainage | Previous treatment failure | *Staphylococcus aureus* | Unknown | 1,500 mg + 1,500 mg/2 weeks (2 doses) | Relapse |
| 77 | Male | ABSSSI  (Wound infection) | Community-acquired | Surgical drainage | Easy use with other pathologies | *Enterococcus faecium* | Three treatments used for 27 days: piperacillin/tazobactam (15), levofloxacin (4), linezolid (8) | 1,500 mg as single dose  Concomitant antimicrobial therapy (linezolid) | Relapse |
| 81 | Male | Osteoarticular infection  (Prosthetic infections) | Health care | Prosthesis removal | Early patient discharge | MSSA | Five treatments used for 43 days: piperacillin/tazobactam (7), amoxicillin/ac clavulanic (14), moxifloxacin (14), vancomycin (4), daptomycin (4) | 1,500 mg + 1,500 mg/week (1 dose) | Reappearance of the clinic of the infection |
| 85 | Male | Osteoarticular infection  (Non prosthetich infection: septic arthritis) | Community-acquired | Surgical drainage | Early patient discharge | MRSA | Four treatments used for 38 days: amoxicillin/ac clavulanic (2), ceftaroline (12), daptomycin (12), ertapenem (12) | 1,500 mg + 1,500 mg/week (1 dose) | Relapse |
| 83 | Male | Osteoarticular infection  (Prosthetic infections) | Health care | No | Previous treatment failure (levofloxacin) | *Staphylococcus epidermidis* | Two treatments used for 72 days: linezolid (24), levofloxacin (48) | 1,000 mg as a single dose  Concomitant antimicrobial therapy | Absence of significant clinical improvement of the infection |
| 82 | Male | Osteoarticular infection  (Prosthetic infections) | Health care | No | Early patient discharge | *Staphylococcus epidermidis* | Two treatments used for 111 days: rifampicin (90), Tedizolid (21) | 1,500 mg as a single dose  Concomitant antimicrobial therapy | Relapse |
| 49 | Female | ABSSSI  (Wound infection) | Community-acquired | No | Previous treatment failure (unkown) | *Staphylococcus epidermidis* and *Enterococcus faecalis* | Unkown | 1,000 mg as a single dose | Recurrence of the signs of infection and bacterial superinfection by another infectious agent |
| 85 | Male | Cardiovascular infection  (Right-sided endocarditis) | Health care | No | Early patient discharge | *Staphylococcus hominis* | Two treatments used for 28 days: vancomycin (14), rifampicin (14) | 1,500 mg as a single dose | Relapse |
| 80 | Female | Osteoarticular infection  (Prosthetic infections) | Health care | Prosthesis removal | Previous treatment failure | *Staphylococcus epidermidis* *Enterococcus faecalis* | Two treatments used for 112 days: daptomycin (56), tedizolid (56) | 1,500 mg as a single dose | Relapse |
| 57 | Female | Catheter-associated infection  (Uncomplicated bacteremia) | Health care | No | Easy use with other pathologies | C*oagulase-negative staphylococci* | Daptomycin was used for 4 days | 1,500 mg as a single dose  Concomitant antimicrobial therapy (daptomycin) | Relapse |

ABSSSIs, acute bacterial skin and skin-structure infections

**Table S3**. Reason for use, type of infection, micro-organism, effectiveness, and safety in patients without prior antibiotic therapy.

|  | **Patients without prior antibiotic therapy** (n=10) |
| --- | --- |
| **Reason for use, n (%)** | |
| Early patient discharge | 7 (70.0) |
| Others/Unknown | 3 (30.0) |
| **Type of infection, n (%)** | |
| Osteoarticular infection | 3 (30.0) |
| Prosthetic infections | 2 (66.7) |
| Non prosthetic infections | 1 (33.3) |
| ABSSSI | 4 (40.0) |
| Cellulitis/Erysipelas | 1 (25.0) |
| Infection of wounds | 2 (50.0) |
| Major cutaneous abscess | 1 (25.0) |
| Cardiovascular infection | 2 (20.0) |
| Right-sided endocarditis | 1 (50.0) |
| Left-sided endocarditis | 1 (50.0) |
| Catheter-related bloodstream infection | 1 (10.0) |
| Uncomplicated bacteremia | 1 (100.0) |
| **Micro-organism, n (%)** | |
| *Staphylococcus aureus* | 4 (40.0) |
| Methicillin-susceptible | 2 (50.0) |
| Unknown | 2 (50.0) |
| Coagulase-negative *staphylococci* | 1 (10.0) |
| *Staphylococcus epidermidis* | 1 (100.0) |
| Enterococci | 1 (10.0) |
| *Enterococcus faecium* | 1 (100.0) |
| Vancomycin-susceptible | 1 (100.0) |
| Polymicrobial | 2 (20.0) |
| Other micro-organisms/ Unknown | 2 (20.0) |
| **Efficacy, n (%)** | |
| Clinical response | 8 (80.0) |
| Clinical failure | 2 (20.0) |
| Relapse | 1 (10.0) |
| **Safety, n (%)** | |
| Adverse events | 0 (0.0) |
| Discontinuation due to adverse events | 0 (0.0) |

ABSSSIs, acute bacterial skin and skin-structure infections

**Table S4**. Reason for use, type of infection, micro-organism, effectiveness, and safety considering patients’ characteristics

|  | **Diabetes**  **mellitus**  (n=44) | **Cardiovascular disease**  (n=51) | **Patients over 60 years** (n=113) | **Patients with no comorbidities at baseline** (n=27) |
| --- | --- | --- | --- | --- |
| **Reason for use, n (%)** | | | | |
| Early patient discharge | 34 (77.3) | 34 (66.7) | 77 (68.1) | 14 (51.9) |
| Previous treatment failure | 1 (2.3) | 6 (11.8) | 9 (8.0) | 6 (22.2) |
| Adverse reaction of previous treatment | 2 (4.6) | 2 (3.9) | 5 (4.4) | 2 (7.4) |
| Others/Unknown | 7 (15.9) | 9 (17.7) | 22 (19.5) | 5 (18.5) |
| **Type of infection, n (%)** | | | | |
| Osteoarticular infection | 13 (29.5) | 6 (11.8) | 37 (32.7) | 13 (48.2) |
| Prosthetic infections | 5 (38.5) | 3 (50.0) | 25 (67.6) | 9 (69.2) |
| Non prosthetic infections | 8 (61.5) | 3 (50.0) | 12 (32.4) | 4 (30.8) |
| ABSSSI | 8 (18.2) | 11 (21.6) | 27 (23.9) | 4 (14.8) |
| Cellulitis/Erysipelas | 1 (12.5) | 2 (18.2) | 6 (22.2) | 1 (25.0) |
| Infection of wounds | 5 (62.5) | 7 (63.6) | 17 (63.0) | 3 (75.0) |
| Major cutaneous abscess | 2 (25.0) | 2 (18.2) | 4 (14.8) | 0 (0.0) |
| Cardiovascular infection | 11 (25.0) | 26 (51.0) | 25 (22.1) | 5 (18.5) |
| Right-sided endocarditis | 3 (27.3) | 7 (26.9) | 7 (28.0) | 2 (40.0) |
| Left-sided endocarditis | 8 (72.7) | 18 (69.2) | 17 (68.0) | 3 (60.0) |
| Vascular prosthetic infections | 0 (0.0) | 1 (3.9) | 1 (4.0) | 0 (0.0) |
| Catheter-related bloodstream infection | 5 (11.4) | 4 (7.8) | 13 (11.5) | 3 (11.1) |
| Uncomplicated bacteremia | 4 (80.0) | 2 (50.0) | 8 (61.5) | 2 (66.7) |
| Complicated bacteremia | 1 (20.0) | 2 (50.0) | 5 (38.5) | 1 (33.3) |
| Other infections | 7 (15.9)^1^ | 4 (7.8)^5^ | 11 (9.7)^9^ | 2 (7.4)^14^ |
| **Micro-organism, n (%)** | | | | |
| *Staphylococcus aureus* | 13 (29.6) | 17 (33.3) | 35 (31.0) | 9 (33.3) |
| Methicillin-susceptible | 8 (61.5) | 7 (41.2) | 15 (42.9) | 6 (66.7) |
| Methicillin-resistant | 5 (38.5) | 7 (41.2) | 15 (42.9) | 2 (22.2) |
| Unknown | 0 (0.0) | 3 (17.6) | 5 (14.3) | 1 (11.1) |
| Coagulase-negative *staphylococci* | 12 (27.3) | 16 (31.4) | 37 (32.7) | 11 (40.7) |
| *Staphylococcus epidermidis* | 8 (66.7) | 12 (75.0) | 25 (67.6) | 10 (90.9) |
| *Staphylococcus hominis* | 2 (16.7) | 1 (6.3) | 3 (8.1) | 0 (0.0) |
| *Staphylococcus lugdunensis* | 0 (0.0) | 0 (0.0) | 1 (2.7) | 0 (0.0) |
| *Staphylococcus haemolyticus* | 1 (8.3) | 0 (0.0) | 0 (0.0) | 1 (9.1) |
| *Staphylococcus capitis* | 0 (0.0) | 1 (6.3) | 1 (2.7) | 0 (0.0) |
| *Staphylococcus pasteuri* | 1 (8.3) | 1 (6.3) | 1 (2.7) | 0 (0.0) |
| Not specified | 0 (0.0) | 1 (6.3) | 6 (16.2) | 0 (0.0) |
| Enterococci | 7 (15.9) | 7 (13.7) | 18 (15.9) | 3 (11.1) |
| *Enterococcus faecalis* | 4 (57.1) | 4 (57.1) | 9 (50.0) | 2 (66.7) |
| *Enterococcus faecium* | 3 (42.9) | 3 (42.9) | 9 (50.0) | 1 (33.3) |
| Vancomycin-susceptible | 7 (100.0) | 5 (100.0)^6^ | 15 (100.0)^10^ | 2 (100.0)^15^ |
| Polymicrobial | 6 (13.6) | 0 (0.0) | 11 (9.7) | 2 (7.4) |
| Other micro-organisms/ Unknown | 6 (13.6)^2^ | 11 (21.6)^7^ | 12 (10.6)^11^ | 2 (7.4)^16^ |
| **Efficacy, n (%)** | | | | |
| Clinical response | 39 (88.6) | 47 (94.0)^19^ | 103 (91.2) | 26 (96.3) |
| Clinical failure | 5 (11.4) | 3 (6.0)^19^ | 10 (8.8) | 1 (3.7) |
| Relapse | 4 (9.1) | 2 (3.9) | 7 (6.2) | 1 (3.7) |
| **Safety, n (%)** | | | | |
| Adverse events | 1 (2.3)^3^ | 2 (3.9)^8^ | 1 (0.9)^12^ | 2 (7.4)^17^ |
| Exitus | 2 (4.5)^4^ | 4 (7.8)^4^ | 10 (8.9)^4^ | 0 (0.0) |
| Discontinuation due to adverse events | 0 (0.0) | 0 (0.0) | 2 (1.8)^13^ | 1 (3.7)^18^ |

ABSSSI, acute bacterial skin and skin-structure infections

^1^ Bacteriemia (n=3), cholangitis (n=2), unknown (n=1), necrotizing fasciitis perianal (n=1); ^2^ *Streptococcus agalactiae* (n=1), *Streptococcus gallolyticus* (n=2), *Streptococcus gordonii* (n=1), unknown (n=2); ^3^ Asthenia (1); ^4^ All exitus were not related with dalbavancin; ^5^ Bacteriemia (n=1), unknown (1), urinary tract infection (n=1), aortic dissection (n=1); ^6^ No antibiogram performed in 2 patients; ^7^ Gram-positive Cocci (n=1), *Corynebacterium jeikeium* (n=1), *Propionibacterium acnes* (n=1), *Streptococcus gallolyticus* (n=1), *Streptococcus gordonii* (n=2), *Streptococcus intermedius* (n=1), unknown (n=4); ^8^ Asthenia (1), balanitis (1); ^9^ Bacteriemia (n=4), urinary tract infection (n=2), intraabdominal abscess (n=1), cholangitis (n=1), unknown (n=2), necrotizing fasciitis perianal (n=1); ^10^ No Antibiogram performed in 3 patients; ^11^ Gram-positive Cocci (n=1), *Propionibacterium acnes* (n=1), *Streptococcus agalactiae* (n=1), *Streptococcus* *gallolyticus* (n=1), *Streptococcus* *gordonii* (n=1), *Streptococcus* *intermedius* (n=1), *Streptococcus* *oralis* (n=1), unknown (n=5); ^12^ Testicular edema (n=1); ^13^ Testicular edema (n=1), severe thrombopenia (n=1); ^14^ urinary tract infection (n=1), pyomyositis (n=1); ^15^ No antibiogram performed in 1 patient; ^16^ Gram-positive Cocci (n=1), *cutibacterium acnes* (n=1); ^17^ Testicular edema (n=1), dizziness (n=1); ^18^ Testicular edema (n=1).^19^ One patients was not evaluable for efficacy

**Table S5**. Reason for use, type of infection, micro-organism, effectiveness, and safety considering the type of micro-organism

|  | **Patients with *Staphylococcus***  ***aureus* infection**  (n=64) | **Patients with Enterococci**  **infection**  (n=24) |
| --- | --- | --- |
| **Reason for use, n (%)** | | |
| Early patient discharge | 43 (67.2) | 14 (58.3) |
| Previous treatment failure | 8 (12.5) | 3 (12.5) |
| Adverse reaction of previous treatment | 2 (3.1) | 2 (8.3) |
| Others/Unknown | 11 (17.2) | 5 (20.8) |
| **Type of infection, n (%)** | | |
| Osteoarticular infections | 20 (31.3) | 4 (16.7) |
| Prosthetic joint infections | 11 (55.0) | 3 (75.0) |
| Non prosthetic infections | 9 (45.0) | 1 (25.0) |
| ABSSSIs | 12 (18.8) | 3 (12.5) |
| Cellulitis/Erysipelas | 4 (33.3) | 0 (0.0) |
| Infection of wounds | 5 (41.7) | 3 (100.0) |
| Major cutaneous abscess | 3 (25.0) | 0 (0.0) |
| Cardiovascular infection | 10 (15.6) | 5 (20.8) |
| Right-sided endocarditis | 3 (30.0) | 0 (0.0) |
| Left-sided endocarditis | 7 (70.0) | 5 (100.0) |
| Catheter-related infection | 15 (23.4) | 4 (16.7) |
| Uncomplicated bacteremia | 10 (66.7) | 2 (50.0) |
| Complicated bacteremia | 5 (33.3) | 2 (50.0) |
| Other infections | 7 (10.9)^1^ | 8 (33.3)^5^ |
| **Micro-organism, n (%)** | | |
| *Staphylococcus aureus* | 64 (100.0) | NA |
| Methicillin-susceptible | 38 (59.4) | NA |
| Methicillin-resistant | 20 (31.3) | NA |
| Unknown | 6 (9.4) | NA |
| Enterococci | NA | 24 (100.0) |
| *Enterococcus faecalis* | NA | 13 (54.2) |
| *Enterococcus faecium* | NA | 11 (45.8) |
| Vancomycin-susceptible | NA | 20 (100.0)^6^ |
| **Efficacy, n (%)** | | |
| Clinical response | 59 (92.2) | 22 (91.7) |
| Clinical failure | 5 (7.8) | 2 (8.3) |
| Relapse | 4 (6.3) | 2 (8.3) |
| **Safety, n (%)** | | |
| Adverse events | 2 (3.1)^2^ | 0 (0.0) |
| Exitus | 3 (4.7)^3^ | 2 (8.3)^3^ |
| Discontinuation due to adverse events | 1 (1.6)^4^ | 0 (0.0) |

ABSSSI, acute bacterial skin and skin-structure infections; NA, not available

^1^ Bacteriemia (n=2), urinary tract infection (n=1), retroperitoneal abscess (n=1), unknown (n=1), aortic dissection (n=1), pyomyositis (n=1); ^2^ Pruritus (n=1), balanitis (n=1); ^3^ All exitus were not related with dalbavancin; ^4^ Severe thrombopenia (n=1); ^5^ Bacteriemia (n=3), urinary tract infection (n=2), Cholangitis (n=2), intrabdominal abscess (n=1); ^6^ No antibiogram performed in 4 patients.
